# The Role of Cathepsin B in the Degradation of Aβ and in the Production of Aβ Peptides Starting With Ala2 in Cultured Astrocytes

**DOI:** 10.3389/fnmol.2020.615740

**Published:** 2021-01-12

**Authors:** Timo Jan Oberstein, Janine Utz, Philipp Spitzer, Hans Wolfgang Klafki, Jens Wiltfang, Piotr Lewczuk, Johannes Kornhuber, Juan Manuel Maler

**Affiliations:** ^1^Department of Psychiatry and Psychotherapy, Friedrich-Alexander-University of Erlangen-Nuremberg, Erlangen, Germany; ^2^Department of Psychiatry and Psychotherapy, University Medical Center, Georg-August-University, Göttingen, Germany; ^3^German Center for Neurodegenerative Diseases, Göttingen, Germany; ^4^Neurosciences and Signaling Group, Department of Medical Sciences, Institute of Biomedicine, University of Aveiro, Aveiro, Portugal; ^5^Department of Neurodegeneration Diagnostics and Department of Biochemical Diagnostics, University Hospital of Bialystok, Bialystok, Poland

**Keywords:** Alzheimer's disease, amyloid beta, cathepsin B, N-terminus, astrocytes, lysosomal

## Abstract

Astrocytes may not only be involved in the clearance of Amyloid beta peptides (Aβ) in Alzheimer's disease (AD), but appear to produce N-terminally truncated Aβ (Aβ_n−x_) independently of BACE1, which generates the N-Terminus of Aβ starting with Asp1 (Aβ_1−x_). A candidate protease for the generation of Aβ_n−x_ is cathepsin B (CatB), especially since CatB has also been reported to degrade Aβ, which could explain the opposite roles of astrocytes in AD. In this study, we investigated the influence of CatB inhibitors and the deletion of the gene encoding CatB (CTSB) using CRISPR/Cas9 technology on Aβ_2−x_ and Aβ_1−x_ levels in cell culture supernatants by one- and two-dimensional Urea-SDS-PAGE followed by immunoblot. While the cell-permeant inhibitors E64d and CA-074 Me did not significantly affect the Aβ_1−x_ levels in supernatants of cultured chicken and human astrocytes, they did reduce the Aβ_2−x_ levels. In the glioma-derived cell line H4, the Aβ_2−x_ levels were likewise decreased in supernatants by treatment with the more specific, but cell-impermeant CatB-inhibitor CA-074, by CA-074 Me treatment, and by CTSB gene deletion. Additionally, a more than 2-fold increase in secreted Aβ_1−x_ was observed under the latter two conditions. The CA-074 Me-mediated increase of Aβ_1−x_, but not the decrease of Aβ_2−x_, was influenced by concomitant treatment with the vacuolar H^+^-ATPase inhibitor Bafilomycin A1. This indicated that non-lysosomal CatB mediated the production of Aβ_2−x_ in astrocytes, while the degradation of Aβ_1−x_ seemed to be dependent on lysosomal CatB in H4 cells, but not in primary astrocytes. These findings highlight the importance of considering organelle targeting in drug development to promote Aβ degradation.

## Introduction

Alzheimer's disease (AD) is the most common neurodegenerative disease in the elderly with neuritic plaques and the neurofibrillary tangles as its neuropathological hallmarks (Glenner and Wong, 1984; Masters et al., 1985; Delacourte and Defossez, 1986; Grundke-Iqbal et al., 1986). The major protein compound of neurofibrillary tangles is hyperphosphorylated tau protein, whereas in neuritic plaques, Amyloid beta (Aβ) peptides represent the predominant protein compound. Aβ peptides are generated from the amyloid precursor protein (APP) by consecutive proteolytic cleavages by β- and γ-secretase and appear to be part of the physiological cell metabolism (Haass and Selkoe, 1993). The most extensively studied β-secretase is the beta-site APP cleaving enzyme 1 (BACE1), which generates the N-terminus of Aβ 1-x by cleaving APP between methionine (671) and aspartic acid (672) (APP770 numbering) (Hussain et al., 1999; Vassar et al., 1999). The highest BACE 1 activity is commonly found in neurons, which seem to be the major source of Aβ 1-x in the central nervous system (Vassar et al., 1999; Lee et al., 2003; Oberstein et al., 2015). However, a major fraction of the Aβ peptides in neuritic plaques in AD brains does not start with the canonical L-aspartic acid residue (Asp1), but is N-terminally truncated or modified (Aβ n-x). These Aβ variants include e.g., Aβ starting with isoaspartate (Aβ 1isoD-x), Aβ starting at Glu3, which is eventually cyclized to pyroglutamate (Aβ 3pE-x), and truncated Aβ peptide variants starting with Ala2, Phe4, and Arg5 (Glenner and Wong, 1984; Masters et al., 1985; Miller et al., 1993; Saido et al., 1995, 1996; Guntert et al., 2006; Bayer and Wirths, 2014). At present, it is not clear how exactly the different N-terminally modified or truncated Aβ variants, that have been detected in neuritic plaques, are generated and which cell types or proteolytic enzymes are involved. An imbalance between the production and degradation of Aβ as well as a shift toward increased proportions of more amyloidogenic Aβ variants via different proteases may promote cerebral Aβ accumulation and amyloid plaque formation (Selkoe, 1998). Aβ variants with truncated N-termini, in particular Aβ variants starting with pyroglutamic acid (Aβ N3pE), and those Aβ variants ending at Ala (42) (Aβ x-42) tend to be more hydrophobic and more amyloidogenic than e.g., Aβ 1-40 and 1-38, which are the most abundant Aβ variants in cerebrospinal fluid, blood plasma and cell culture supernatants (Haass and Selkoe, 1993; Pike et al., 1995; Wang et al., 1996; Thal et al., 2006; Bayer and Wirths, 2014; Oberstein et al., 2015; Schonherr et al., 2016). The vascular deposits do not possess a dense core primarily made of Ab x-42 like the parenchymal neuritic or senile plaques (Thal et al., 2006). They contain mainly Aβ x-40 (Glenner and Wong, 1984; Akiyama et al., 1997). The N-terminally truncated Aβ 2-x peptides were found in particular in parenchymal and vascular amyloid deposits in AD brains (Wiltfang et al., 2001; Schieb et al., 2011; Savastano et al., 2016; Wildburger et al., 2017; Zampar et al., 2020). Aβ 2-40 seemed to be elevated in AD cases with severe cerebral amyloid angiopathy (CAA) compared to AD cases without CAA (Gkanatsiou et al., 2019). A potential source for these N-terminally modified Aβ may be reactive astrocytes and microglia, as they are located in the immediate vicinity of neuritic plaques. They seem to be involved in changes in the amyloid plaque composition by means of ineffective phagocytosis, secretion of proteases, and interactions with the peripheral immune system (Selkoe, 2001; Nagele et al., 2003; Thal et al., 2006). In previous studies we have shown that the role of astrocytes and microglia in AD may not be limited to Aβ plaque removal or modification: In cell culture, these cells secrete higher proportions of N-terminally modified or truncated Aβ variants like Aβ 2/3-40 and Aβ 4/5-40 in relation to Aβ 1-x than neuronal cells (Oberstein et al., 2015). The cellular production of the presumed Aβ 2-40 variant was found to be independent of BACE1. A number of different candidate proteases, such as cathepsin B (CatB), meprin β, neprilysin, myelin basic protein, the metalloproteinase ADAM TS4 or aminopeptidases, have been proposed to act in cooperation with or independently of BACE1 to produce these N-terminally modified Aβ variants (Howell et al., 1995; Saido, 1998; Hook et al., 2005; Liao et al., 2009; Sevalle et al., 2009; Bien et al., 2012; Bayer and Wirths, 2014; Walter et al., 2019). In this study, we chose to investigate the cysteine protease CatB for its ability to generate Aβ in astrocytes, because assays from cell extracts and purified secretory vesicles indicated that CatB exerts β-secretase activity and thereby promotes the production of Aβ (Hook et al., 2005; Bohme et al., 2008; Schechter and Ziv, 2011). On the other hand, CatB seemed to degrade Aβ via C-Terminal truncation, leaving its role for the Aβ metabolism unclear (Mackay et al., 1997; Mueller-Steiner et al., 2006). Accordingly, both protective and deleterious effects of CatB on memory loss and Aβ plaque load have been described (Mueller-Steiner et al., 2006; Sun et al., 2008; Hook et al., 2010, 2011; Kindy et al., 2012; Moon et al., 2016; Embury et al., 2017). In general, CatB seems to be involved in cell cycle regulation, the pathophysiology of multiple cancers, autophagy and neuroinflammation (Yan and Sloane, 2003; Chai et al., 2019). CatB has also been linked to a plethora of other diseases of the central nervous system, including AD, intracerebral hemorrhages, and traumatic brain injury (Cataldo and Nixon, 1990; Hook et al., 2005, 2014; Mueller-Steiner et al., 2006; Kindy et al., 2012). In AD, elevated levels of CatB have been detected in brains of AD patients extracellularly near neuritic plaques in membrane bound organelles, in degenerating neuronal perikarya, and in reactive astrocytes (Cataldo and Nixon, 1990; Cataldo et al., 1991; Nakamura et al., 1991). Elevated CatB activity in plasma samples of AD patients has been published (Sundelof et al., 2010; Morena et al., 2017). CatB has both endopeptidase and exopeptidase activities (Taralp et al., 1995). Under physiological conditions, CatB is mainly active in early endosomes and lysosomes (Taralp et al., 1995). At low pH, it exerts a high carboxypeptidase activity on APP and Aβ in cell free assays (Mackay et al., 1997). Tumor cells secrete CatB into extracellular space, where it is stabilized by heparin sulfate on the plasma membrane, and its endopeptidase activity is favored due to neutral pH values (Almeida et al., 2001; Cotrin et al., 2004). To investigate whether CatB was involved in the generation of Aβ 1-x and the N-terminally truncated Aβ 2-x variants by astrocytes, we tested the cysteine protease inhibitor E64d and the CatB inhibitor CA-074 Me on chicken and human primary astrocytes. We selected the chicken as a model organism, because the Aβ amino acid sequence is the same as in humans. To further confirm the observed effects of the CA-074 Me treatment the gene encoding CatB (CTSB) was deleted via CRISPR/Cas 9 technology in the glioma derived cell line H4. The patterns of Aβ variants in the conditioned cell culture media were assessed by one- (1D) and two-dimensional (2D) Urea-SDS-PAGE followed by Western blotting.

## Materials and Methods

### Isolation and Cultivation of Primary Cells

Chicken neurons and astrocytes from specific pathogen free eggs (Valo Biomedia, Osterholz-Scharmbeck, Germany) were prepared and cultivated as previously described. Human fetal astrocytes (provitro/Sciencell, Berlin, Germany) were cultivated as previously described (Oberstein et al., 2015).

### Cultivation of Cell Lines

Untransfected human brain neuroglioma H4 cells (H4, LGC Standards GmbH/ATCC, Wesel, Germany), H4 cells stably transfected with human APP 751 (H4 APP 751) or H4 APP 751 (see below) with and without bi-allelic deletion of CTSB via CRISPR/Cas9 (H4 APP754 CTSB –/–) were maintained in DMEM medium supplemented with 10% superior fetal bovine serum (FBS, Biochrom, Berlin, Germany), 100 IU/ml penicillin and 100 μg/ml streptomycin (Biochrom) with or without 500 μg/ml G418 (Thermo Fisher Scientific/Roche, Grenzach-Wyhlen, Germany) and/or 2 μg/ml Puromycin (Santa Cruz Biotechnology, Heidelberg, Germany) respectively. A complete change of the medium was performed every two to three days. For the assessment of Aβ in conditioned supernatants, the medium was changed to serum-free DMEM/Ham's F12 (Biochrom) with G5 supplement (Thermo Fisher Scientific/Gibco, Darmstadt, Germany) and 10 mM Hepes (Biochrom).

### Drug Treatment and Sample Preparation

The cysteine protease inhibitor E64d (100 mM, Peptanova, Sandhausen, Germany), the H^+^-ATPase inhibitor Bafilomycin A1 (20 μM, Sigma Aldrich, Munich, Germany) the cathepsin B inhibitors CA-074 Me (25 mM, Peptanova), and CA-074 (25 mM, Sigma Aldrich, Munich, Germany) were dissolved in dimethyl sulphoxide (DMSO, Carl Roth, Karlsruhe, Germany) and stored at −20 °C. For the analysis of the released Aβ, the expression of APP, of BACE1, and of CatB, a complete medium change with serum-free medium was performed prior to treatment with drugs or with DMSO alone, yielding maximum final concentrations of 0.2% v/v DMSO. The cells were treated over 48 h. The conditioned media were subsequently centrifuged at 500 g for 5 min and stored at −20°C. Cells were washed with phosphate buffered saline (PBS, Biochrom) for 5 min at room temperature (RT) and lysed in detergent buffer [50 mM HEPES, 0.037 w/v Complete Mini Protease Inhibitor Cocktail (Thermo Fisher Scientific/Roche), 150 mM NaCl, 1% v/v Non-idet P-40, 0.5% w/v sodium deoxycholate, and 0.1% w/v sodium dodecylsulfate (SDS)] or in CytoBuster™ Protein Extraction Reagent (Merck Millipore) for 10 min at 4°C. The lysed cells were centrifuged (13,000 g, 5 min, 4°C), and supernatants were stored at −70°C.

### Transfection of APP 751 Into H4 Cells

Full-length cDNA of human amyloid beta A4 protein isoform b precursor (also known as PreA4 751, APP 751) cloned into a pCI-neo mammalian expression vector (Promega, Mannheim, Germany) was kindly provided by Prof. Dr. Oliver Wirths (University Medical Center Goettingen, Goettingen). Twenty four hours post-seeding, the construct (7.5 μg / 9.5 cm^2^ growth area) was transfected in 70% confluent H4 neuroglioma cells by calcium phosphate co-precipitation in serum-free medium. After 48 h, G418 (Thermo Fisher Scientific/Roche) resistant clones were selected by limiting dilution at <0.2 cells/well in DMEM with 10%FBS 100 IU/ml penicillin, 100 μg/ml streptomycin, 500 μg/ml G418 and maintained in the presence of G418. Six clones were isolated and assessed for the level of APP expression in 1D Aβ-PAGE.

### Knockout of CTSB in H4 APP 751 wt Cells *via* CRISPR/Cas9

CTSB –/– cell lines were generated from H4 cells and H4 APP 751 cells using CRISPR/Cas9 KO and HDR Plasmid (Santa Cruz Biotechnology) according to the protocol of the supplier. Empty CRISPR/Cas9 plasmids (Santa Cruz Biotechnology) were used as control. For generation of single-cell colonies, Puromycin (Santa Cruz Biotechnology) resistant clones were selected by limiting dilution at <0.2 cells/well and maintained in the presence of 2 μg/ml puromycin. Five clones of H4 and two clones of H4 APP 751 cells were identified having a bi-allelic knockout for CTSB by western blot analysis.

### Cell Viability Assays

Cell viability was assessed after drug treatment and knockout using the CytoTox 96® lactate dehydrogenase (LDH) assay (Promega) according to the manufacturer's instructions and for cells treated with CA-074 Me or E64d using the MTT [3-(4,5-dimethylthiazol-2-yl)-2,5-diphenyltetrazolium bromide] assay, as previously described (Mosmann, 1983).

### Immunoprecipitation of Aβ

For the immunoprecipitation of Aβ, 40 μg mouse anti- Aβ n-x 6E10 (mAb 6E10, BioLegend formerly Covance, Koblenz, Germany) were covalently coupled to 10 mg magnetic sheep anti-mouse Dynabeads® M-280 (Dynal, Hamburg, Germany) according to the manufacturer's instructions.

For the detection of N-terminally truncated Aβ, 5, 10, or 20 ml of conditioned cell culture supernatant of chicken astrocytes, human astrocytes or H4 cells were supplemented with Complete Mini Protease Inhibitor Cocktail (Thermo Fisher Scientific/ Roche) and concentrated 5 to 10-fold with 3,000 MWCO Vivaspin Protein Concentrators (GE Healthcare, Munich, Germany) at 4,000 g and 4°C. Conditioned media (with or without prior concentration) were mixed with 5-fold triple detergent buffer concentrate and 25 μl of magnetic beads coupled with mouse anti-Aβ antibody (mAb 6E10); yielding final concentrations of 1 μg/ml of immobilized mAb 6E10 in 50 mM HEPES, 150 mM NaCl, 0.5% v/v Non-idet P-40, 0.25% w/v sodium deoxycholate, and 0.05% w/v SDS. Immunoprecipitation was performed under rotation for 15 h at 4°C. For the analysis with Urea-SDS–PAGE, the samples were rinsed three times with PBS/0.1% BSA for 5 min at 4°C and once with 10 mM Tris-HCl, pH 7.5. For 1D-Aβ-PAGE and for 2D-Aβ-PAGE, the Aβ were eluted as previously described (Maler et al., 2007; Oberstein et al., 2015).

### BCA Assay and Tris/glycine SDS-PAGE (SDS-PAGE) Followed by Western Blot and Immunodetection (IB)

The concentration of total protein in cell lysates was assessed with the bicinchoninic acid assay (BCA assay, Thermo Fisher Scientific/Pierce) as previously described (Smith et al., 1985). The absorption at 562 nm was measured with a Benchmark Microplate Reader (Bio-Rad, München, Germany) and was analyzed with Microplate Manager 5.1 software (Bio-Rad, München, Germany). 0.5 μg of protein sample per lane in sample buffer (63 mM Tris/HCl pH 6,8; 0.5% w/v SDS; 2.5% v/v glycerol; 100 mM w/v dithiothreitol; 0,0125% w/v bromophenol blue) were separated by 25 mM Tris pH 8,3/0,192 M glycine 0.1 w/v% SDS-PAGE with a 4% T/2.67% C stacking gel and a 7.5% T/2.67% C running gel for the detection of APP and a 10% T/2.67% C running gel for the detection of cathepsin B or BACE1 at RT and 200 V constant voltage (Laemmli, 1970). Separated proteins were transferred to Immobilon-FL PVDF membranes (Merck Millipore, Darmstadt, Germany), blocked with 2% w/v Amersham ECL advance blocking agent (GE Healthcare, Munich, Germany), probed with mouse anti-Cathepsin B CA10 (1:400 in PBS/0.1% v/v Tween (PBS-T); abcam, Cambridge, UK), rabbit anti-BACE1 PA1-757 (1:200 in PBS-T, Thermo Fisher Scientific) or mouse anti-APP 22C11 (1:1000 in PBS-T, Merck Millipore). After three times washing for 10 min with PBS-T the blots were incubated for 60 min with horseradish peroxidase (POD) conjugated goat anti-mouse or horse anti-rabbit antibodies (Merck Millipore). Mouse anti-GAPDH 374 (1:5000; Merck/Millipore) served to detect GAPDH as a loading control. Chemiluminescence was recorded after 5 min incubation at room temperature with ECL Prime Western Blotting Detection Reagent (GE Healthcare) with an Amersham Imager 600 (GE Healthcare).

### CatB Activity Assay

CatB activity in cells lysed with the CytoBuster™ Protein Extraction Reagent was assessed with the InnoZyme™ Cathepsin B Activity Assay Kit (Merck Millipore/Calbiochem) according to the supplier's information. The different samples were adjusted to equal protein concentrations according to the results from BCA protein assay. Free AMC was measured using a Victor 3 multilabel plate reader (Perkin Elmer, Rodgau, Germany) with 355 nm excitation and 460 nm emission wavelengths and was quantified with Wallac 1420 software (Perkin Elmer).

### Urea-Bicine/Bis-Tris/Tris/Sulfate SDS-PAGE Followed by Western Blot and Immunodetection

Stock solutions of synthetic peptides Aβ_1−40_, Aβ_2−40_, Aβ_3−40_, Aβ_pE3−40_ and Aβ_4−40_ and Aβ_5−40_ (1 mg/ml, MoBiTec/Anaspec, Goettingen, Germany) were prepared in sample buffer (0.36 M Bis–Tris, 0.16 M Bicine, 15% w/v sucrose, 1% w/v SDS, and 0.0075% w/v bromophenol blue) and stored at −80°C. Aβ levels in cell culture supernatants, were analyzed by urea-bicine/bis-tris/tris/sulfate SDS-PAGE followed by immunoblot as previously described (Klafki et al., 1996; Oberstein et al., 2015). The development of the immunoblots with mouse monoclonal anti-Aβ 1-x 82E1 (mAb 82E1, 1:1000 in PBS-T; IBL) or rabbit polyclonal anti-Aβ 2-x p77 (Savastano et al., 2016) was performed as previously described (Oberstein et al., 2015; Savastano et al., 2016).

### 2D Urea-SDS-PAGE and Immunoblot

Two-dimensional electrophoretic separation of Aβ peptides and immunoblot analysis were performed as previously described (Maler et al., 2007; Oberstein et al., 2015). The procedure for immunodetection with different anti-Aβ antibodies was as described above.

### Organelle Stains

LysoTracker® as an organelle dye was used to examine the effects of chemical protease inhibitors and deletion of CatB on the morphology of lysosomes. Human astrocytes and H4 CTSB ± and CTSB –/– cells, were incubated with 75 nM working concentration of LysoTracker® Red DND-99 (Thermo Fisher) at 37°C for 30 min according to the manufacturer's instructions. Visualization was performed under a Leica DM IL HC Bio fluorescence microscope (excitation filter: BP 561/14, beamsplitter: BS R561, suppression filter: 609/54). Particle analysis was performed using ImageJ v1.46R.

### Statistical Analysis

The data were analyzed with GraphPad Prism version 6.02 (GraphPad Software, San Diego, CA, USA) and SPSS Statistics version 22.0 (IBM, San Jose, CA, USA). Differences between groups were assessed with unpaired and ratio paired *T*-test, Kruskal-Wallis Test, one-way analysis of variance (ANOVA), and two-way ANOVA followed by Dunnett's or Tukey's multiple comparisons test, as a *post-hoc* test when a significant effect was observed. The degrees of freedom (d.f.) for the associated tests are given in brackets. All data are expressed as the mean ± standard deviation (SD). Significance levels are indicated as follows: ^***^p < 0.001; ^**^p < 0.01; ^*^p < 0.05; and ns, not significant.

## Results

### The Cell Permeant-Cysteine Proteinase Inhibitor E64d and the Cell-Permeant CatB Inhibitor CA-074 Me Reduced the Amount of Aβ 2–40

**Figure 1:**
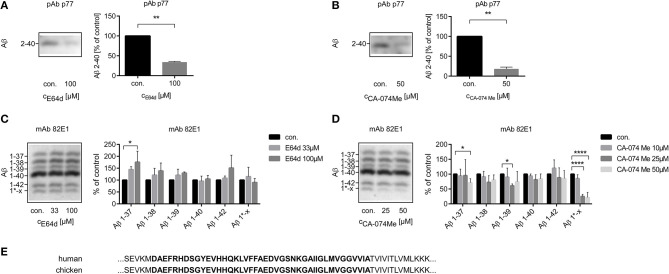
Quantification of the relative amounts of Aβ 2-x **(A,B)** and of Aβ 1-x **(C,D)** in conditioned medium of cultured chicken astrocytes after the treatment with E64d **(A,C)** or CA-074 Me **(B,D)** over 48 h compared to controls using 1D Urea-SDS-PAGE followed by immunoblot analysis with anti-Aβ 2-x polyclonal antibody (pAb) p77 **(A,B)** or anti-Aβ 1-x monoclonal antibody (mAb) 82E1 **(C,D)**. Twenty milliliter **(A,B)** or 4 ml **(C,D)** sample volume were used. Aβ 2-40 was significantly reduced after the treatment with E64d **(A)** or CA-074 Me **(B)**. No significant reduction of the amounts of Aβ 1-40 or Aβ 1-42 was detected after E64d **(C)** or CA-074 Me **(D)** treatment. The labeling of the different Aβ 1-x peptide variants is based on a series of synthetic Aβ (Aβ 1-37, Aβ 1-38, Aβ 1-39, Aβ 1-40, and Aβ 1-42) and their isoelectric point in 2D Urea-SDS-PAGE (Supplementary Figure 1). A specific Aβ variant, designated as Aβ 1*-x, was effectively reduced by CA-074 Me treatment **(D)**. Panel **(E)** shows a section of the amino acid sequences of human and chicken APP, which contains the amino acid sequence of Aβ 1-42 (bold) and adjacent amino acids. In contrast to rodents, no differences in the amino acid sequence exist between humans (NP _958816.1) and chicken (NP _989639.1) in this part of APP (NCBI Reference Sequences are given, http://www.uniprot.org). Statistics: **(A)** *n* = 3, ratio paired *T*-Test *t*_(2)_ = 31.43 *p* < 0.01, **(B)** *n* = 3, ratio paired *T*-Test *t*_(2)_ = 12.05 *p* < 0.01, **(C)** *n* = 3, Kruskal-Wallis test Aβ 1-37, *H* (2) = 5. 793, *p* < 0.05, eta^2^ = 0.63, Kruskal-Wallis test Aβ 1-38, *H* (2) = 3.310, *p* > 0.05, Kruskal-Wallis test Aβ 1-39, *H* (2) = 3.310, *p* > 0.05, Kruskal-Wallis test Aβ 1-40, *H* (2) = 0.8276, *p* > 0.05, Kruskal-Wallis test Aβ 1-42, *H* (2) = 0.1036, *p* > 0.05, Kruskal-Wallis test Aβ1^*^-x, *H* (2) = 1.471, *p* > 0.05, **(D)** *n* = 5, Kruskal-Wallis test Aβ 1-37*, H* (3) = 10.45, *p* < 0.05, eta^2^ = 0.41, Kruskal-Wallis test Aβ 1-38, H (3) = 6.529, *p* > 0.05, Kruskal-Wallis test Aβ 1-39, *H* (3) = 8.236, *p* < 0.05, Kruskal-Wallis test Aβ 1-40, *H* (3) = 6.101, *p* > 0.05, Kruskal-Wallis test Aβ 1-42, *H* (3) = 4.178, *p* > 0.05, Kruskal-Wallis test Aβ 1*-x, *H* (3) = 17.23, *p* < 0.001, eta^2^ = 0.79. Dunn's *post-hoc* test was performed for comparisons to vehicle treated control (con.). Selected comparisons are indicated as follows*p < 0.05; **p < 0.01, and ****p < 0.0001).

The amounts of Aβ 2-x or Aβ 1-x in cell culture supernatants were assessed by 1D Urea-SDS-PAGE followed by immunoblot. Treatment with the irreversible cysteine proteinase inhibitor E64d or with the CatB inhibitor CA-074 Me significantly reduced the amount of Aβ 2-x in conditioned media of cultured chicken astrocytes to 34.2 ± 1.2% and 18.2 ± 2.6 of the respective controls (Figures 1A,B). In contrast, separate immunoblots probed with the monoclonal antibody (mAb) 82E1, which specifically recognizes Aβ 1-x, indicated that the levels of Aβ 1-37, Aβ 1-38, Aβ 1-39, Aβ 1-40, and Aβ 1-42 were not decreased by E64d (Figure 1C). Treatment of cultured chicken astrocytes with high concentrations of CA-074 Me appeared to slightly reduce the levels of Aβ 1-37, Aβ 1-38, and Aβ 1-39 without significantly affecting the amount of Aβ 1-40 or Aβ 1-42 compared to controls (Figure 1D). The separation of Aβ peptide variants by 2D Urea-SDS-PAGE was used to further characterize the detected Aβ 1-x and 2-x variants. 2D-immunoblots probed with mAb p77 showed that the most abundant Aβ 2-x variant in supernatants of cultivated chicken astrocytes had the same pI and electrophoretic mobility in the second dimension as Aβ 2-40. Additionally, Aβ variants co-migrating with Aβ 2-38 and Aβ 2-42 were observed (Supplementary Figure 1). On 1D-immunoblots, Aβ 2-38 and Aβ 2-42 were usually not detected, presumably due to lower analytical sensitivity. Interestingly, a single, specific Aβ variant (designated Aβ^*^ 1-x) was substantially and statistically significantly reduced by CA-074 Me treatment (Figure 1D). The exact length and chemical structure of Aβ^*^ 1-x remains elusive, however, it is recognized by mAb 82E1, which is highly selective for Aβ starting with Asp (1) (Oberstein et al., 2015). On 2D-immunoblots, Aβ^*^ 1-x showed a shifted isoelectric point (pI) of ~6.4, which is substantially different from that of Aβ 1-38, Aβ 1-40, and Aβ 1-42, which all have a pI of ~5.4 (Supplementary Figure 2). In order to find out whether not only the amino acid sequence of Aβ between chickens and humans is identical (Figure 1E), but the CatB inhibition has a similar effect on the processing of APP, cultured human astrocytes were also treated with CA-074 Me (Supplementary Figure 3).

In human astrocytes, data from two individual experiments indicated that the amount of Aβ 2-40 was reduced after 48h incubation with 25 μM CA-074 Me (Supplementary Figure 3A). The level of the highly abundant Aβ 1-40 remained unchanged by CA-74 Me treatment in supernatants of cultured human astrocytes (Supplementary Figure 3B). Aβ 1-37 was significantly increased by 25μM CA-74 Me (169 ± 43% of controls). Aβ 1-37 is generally low abundant in cell culture supernatants, and even after its increase by CA-074Me treatment, its level was only 4.8 ± 2.1% of Aβ 1-40.

Collectively, the treatment with the cell permeant proteinase inhibitors E64d and CA-074 Me reduced the relative amount of Aβ 2-x in cell culture supernatants of primary astrocytes mostly without displaying significant effects on the secretion of Aβ 1 -x (see Table 1).

**Table 1:** Comparison of the effects of 50 μM CA-074 Me, 50 μM CA-074 and 100 μM E64d on the relative abundances of different Aβ peptide variants in supernatants of H4 cells, H4 APP 751 cells, H4 APP 751 CTSB –/– cells, chicken astrocytes, and human astrocytes compared to controls.

**H4APP 751/(H4)**	**Aβ_1−40_**	**Aβ_1−42_**	**Aβ_2−40_**
CA-074 Me (50 μM)	↑↑/(↑)	↑↑	↓
CA-074 (50 μM)	↔	↔	↓
**H4APP 751 CTSB–/–**	**Aβ_1−40_**	**Aβ_1−42_**	**Aβ_2−40_**
CA-074 Me (50 μM)	↔	↔	↔
**Chicken astrocytes/(Human astrocytes)**	**Aβ_1−40_**	**Aβ_1−42_**	**Aβ_2−40_**
E64d	↔	↑	↓↓
CA-074 Me	↔/(↔)	↔/(↔)	↓↓/(↓↓^*^)

The decrease of Aβ 2-40 in supernatants from human astrocytes after CA-074 Me treatment has to be considered as preliminary (n = 2, *).

*x-fold increase: ↑↑ $\underline{\underline{\wedge }}$ x > 200%; ↑ $\underline{\underline{\wedge }}$ 125% < x <200%, ↔ $\underline{\underline{\wedge }}$ 75% < x < 125%; ↓ $\underline{\underline{\wedge }}$ 50% < x < 75%; ↓↓ $\underline{\underline{\wedge }}$ 10% < x < 50%*.

The tested concentrations of E64d or CA-074 Me did not lead to a significant decrease in cell viability or cytotoxicity (data not shown). In cell extracts of cultured chicken astrocytes treated with 25 and 50 μM CA-074 Me, the activity of CatB was below the detection range of the cathepsin B activity assay (Supplementary Figure 4A). The cellular CatB, BACE1 and APP levels in western blot analysis as well as total protein according to BCA assay were not significantly changed after the treatment with 50 μM CA-074 Me for 48 h (Supplementary Figures 4B,C).

### Mature CatB Protein Levels and CatB Activity Were Higher in Cultured Chicken Astrocytes Than in Neurons

**Figure 2:**
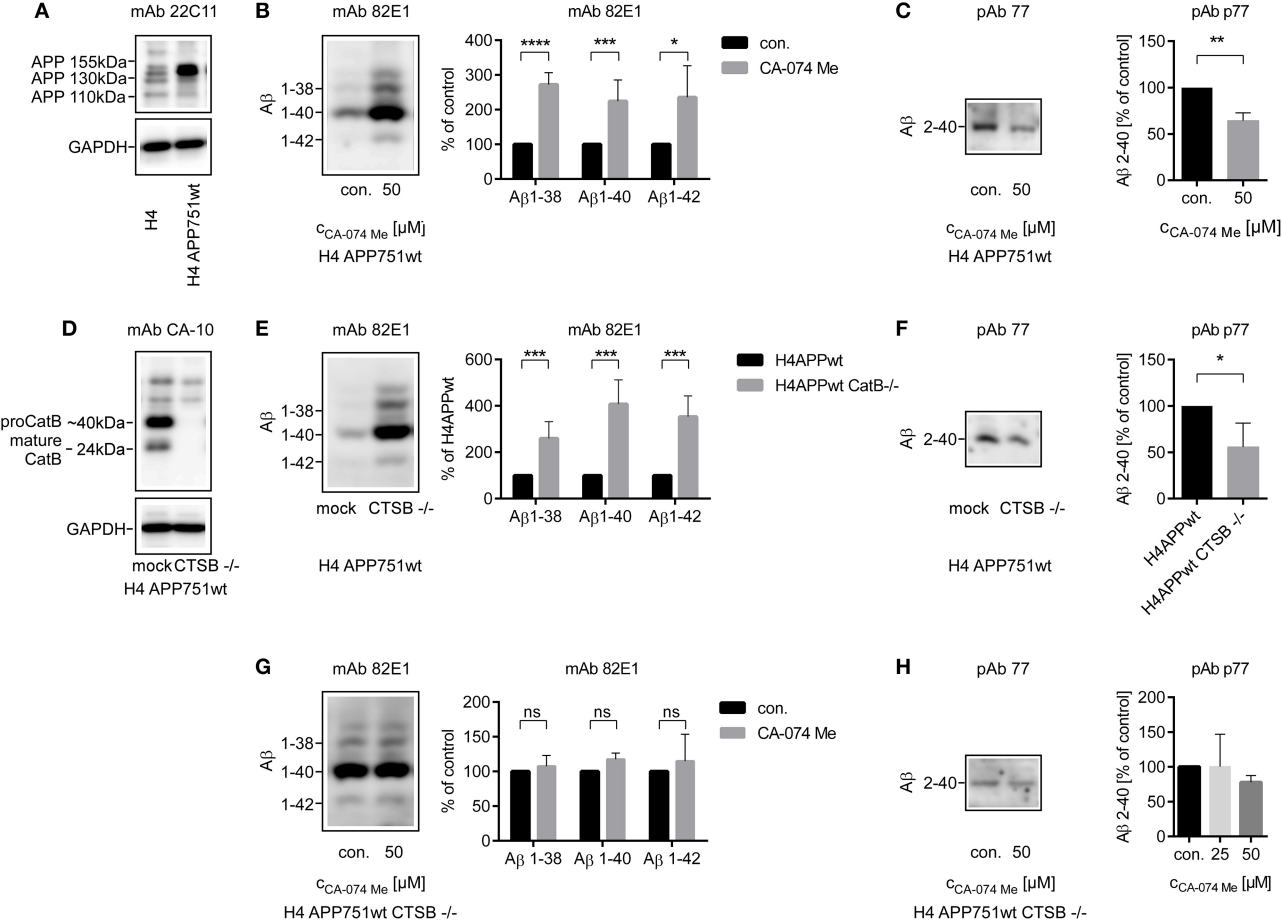
Stable transfection of APP751 in H4 cells was controlled by SOS-PAGE followed by immunoblot probed with anti-APP mAb 22C11 **(A)**. Four milliliters **(B,E,G)** or 10 ml **(C,F,H)** of conditioned media from H4 APP751wt cells were used for the analysis of secreted Aβ peptides. The relative abundance of Aβ 1-x **(B)** was increased after the stimulation with CA-074 Me over 48 h in supernatants of H4 APP751wt cells as assessed by 1D Urea-SDS-PAGE followed by immunoblot, whereas the amount of Aβ co-migrating with Aβ 2-40 was decreased compared to controls **(C)**. Deletion of CTSB *via* CRISPR/Cas9 technology was controlled by SOS-PAGE followed by immunoblot probed with anti-CatB mAb CA-10 **(D)**. The abundance of Aβ 1-x was increased in H4 APP751wt CTSB –/– cells compared to H4 APP751wt cells **(E)**, whereas Aβ co-migrating with Aβ 2-40 was slightly decreased **(F)**. In H4 APP751wt CTSB –/– cells, treatment with CA-074 Me did not significantly alter the abundance of Aβ co-migrating with synthetic Aβ 1-38, Aβ 1-40 or Aβ 1-42 **(G)**, but it slightly reduced the amount of Aβ 2-x **(H)**. Statistics: **(B)** ratio paired *T*-Test Aβ 1-38, *t*_(4)_ = 18.04, *p* < 0.001, **(F)** *n* = 4, ratio paired *T*-Test *t*_(3)_ = 3.435, *p* < 0.05 **(G)** *n* = 3, ratio paired *T*-Test Aβ 1-38, *t*_(2)_ = 0.3416, *p* > 0.05, ratio paired *T*-Test Aβ 1-40, *t*_(2)_ = 3.857, *p* > 0.05, ratio paired *T-*Test Aβ 1-42, *t*_(2)_ = 2.776, *p* > 0.05 **(H)** *n* = 3, Kruskal-Wallis test *H* (2) = 3.310, *p* > 005. Selected comparisons are indicated as follows *p < 0.05; **p < 0.01; ***p < 0.001; and ***p < 0.0001.

CA-074 Me appeared to affect mainly Aβ 2-x peptides, which are typically secreted by astrocytes. Thus, the relative abundance of CatB protein and its enzymatic activity were compared between cultured chicken astrocytes and chicken neurons. Expression of mature CatB, as assessed by western blot, and CatB activity in cell lysates were more than 4-fold higher in astrocytes than in neurons (Supplementary Figures 5A,B). Immature and mature BACE1 proteins (67 and 59 kDa, respectively) were detected in cultured chicken neurons but not in chicken astrocytes by SDS-PAGE followed by immunoblot (Supplementary Figure 5C). Chicken astrocytes predominantly expressed longer isoforms of APP compared to chicken neurons (Supplementary Figure 5D), which was in line with previous reports (Rohan de Silva et al., 1997). Accordingly, the APP 751 isoform and not the shorter APP 695 isoform was chosen for the transfection of cell lines (Figure 2A).

### CRISPR/Cas9 Induced Knockout of CTSB and CatB-Inhibition With CA-074 Me in H4 APP 751 Neuroglioma Cells Increased the Amount of Secreted Total Aβ

Aβ 2-x concentrations are small and cannot be reliably measured by Western blot in primary cell culture experiments with sample volumes that are typically used e.g., in gene silencing experiments. Thus, a H4 neuroglioma cell line stably overexpressing the amyloid precursor protein transcript variant b (APP 751; H4 APP 751) was established, and the effect of a CRISPR/Cas9 induced CTSB knockout (H4 APP 751 CTSB–/–) on Aβ peptide variants in the conditioned media was investigated (Figures 2E–H).

In contrast to the findings in primary cell culture experiments (see above), the concentrations of Aβ 1-38, Aβ 1-40, and Aβ 1-42 in the supernatant of H4 APP 751 cells were increased after treatment with 50 μM CA-074 Me according to Aβ immunoblot analysis (Figure 2B) In sharp contrast, Aβ 2-x was reduced after CA-074 Me treatment (Figure 2C). The increase in Aβ 1-x in conditioned medium after treatment with 25 and 50 μM CA-074 Me was also observed after normalization to total cellular protein levels of H4 APP 751 cells. According to BCA protein assay, the cellular protein levels were slightly increased after CA-074 Me treatment (25 μM: 1.26 ± 0.34 of control and 50 μM: 1.21 ± 0.21 of control). The CatB level in cell lysates of H4 APP 751 cells remained unchanged by CA-074 Me treatment (Supplementary Figure 6).

The CRISPR/Cas9 induced knockout of CTSB in H4 APP 751 cells (H4 APP 751 CTSB –/–; Figure 2D) led to a strong increase in Aβ 1-40, Aβ 1-42, and Aβ 1-38 in supernatants compared to mock treated H4 APP 751 cells (Figure 2E). In contrast, the abundance of Aβ 2-x in supernatants of H4 APP 751 CTSB –/– cells was moderately reduced to 0.55 ± 0.22 in comparison to H4 APP 751 cells (Figure 2F). Treating H4 APP 751 CTSB –/– cells with 50 μM CA-074 Me did not have an additional effect, and thus did not significantly change the amounts of Aβ 1-38, Aβ 1-40, and Aβ 1-42 in conditioned medium compared to controls (Figures 2G,H).

Collectively, the observations after pharmacological inhibition of CatB with CA-074 Me and CatB knockout suggested that CatB was probably involved in the degradation of Aβ in H4 APP 751 cell cultures but not in primary astrocytes (see Table 1). To exclude that this was a consequence of the APP 751 overexpression, non–transfected H4 cells were treated with CA-074 Me. This resulted in a similar increase in the abundance of Aβ 1-40 as observed in H4 APP 751 cells (Supplementary Figure 7).

### Secreted Aβ 1-x Was Reduced by the Cell-Impermeant CatB Inhibitor CA-074 and H^+^-ATPase Inhibitor Baf-A1 in H4 APP 751 Cells

**Figure 3:**
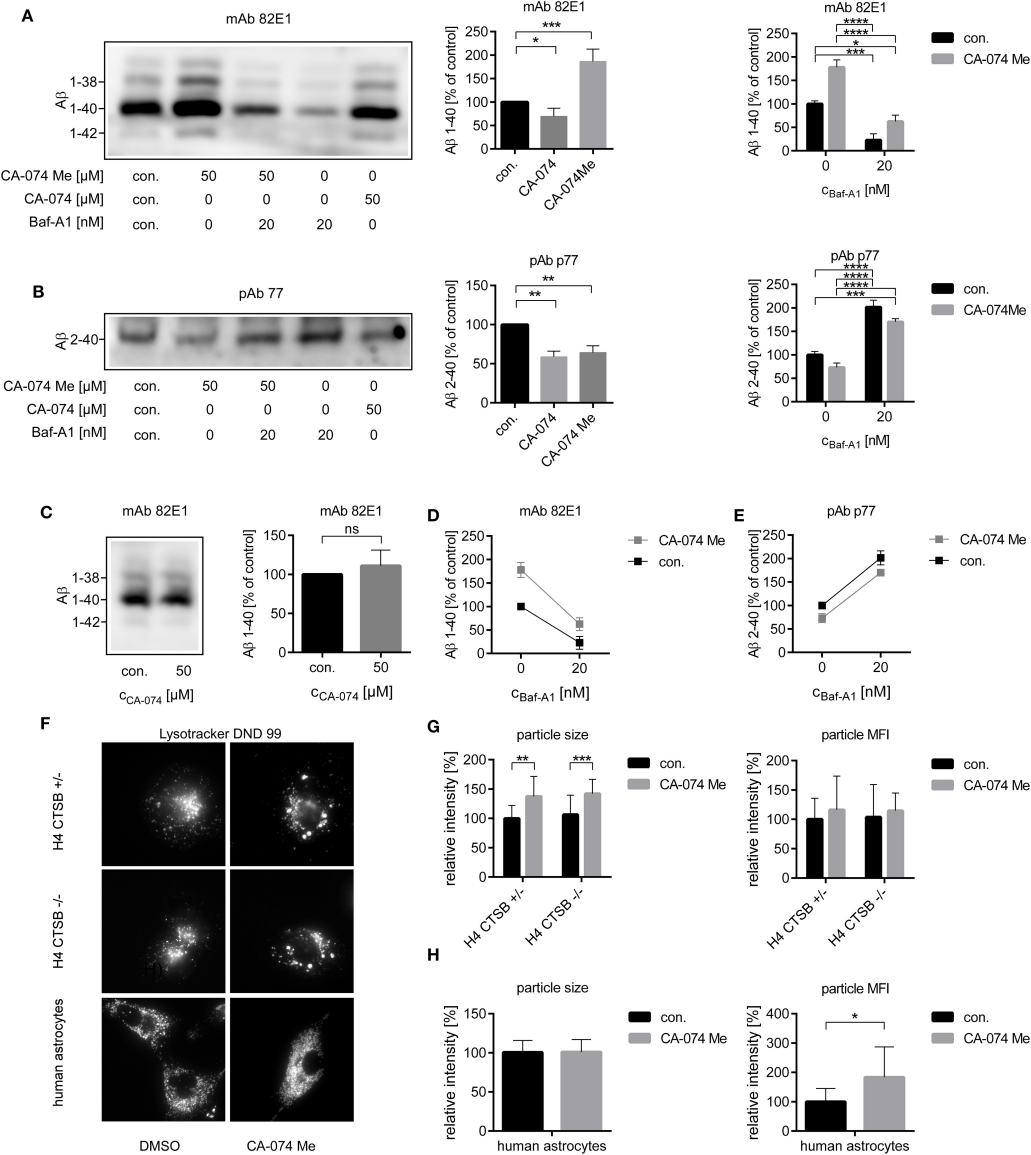
Four milliliters **(A,C,D)** or 20 ml **(B,E)** of conditioned media from H4 APP751 wt cells were used for the analysis of secreted Aβ. The relative amounts of Aβ 1-x **(A)** and of Aβ 2-40 **(B)** were decreased after the treatment of H4 APP741 wt cells with the cell-impermeant CatB-inhibitor CA-074 over 48 h. In supernatants of CA-074 Me treated cells, only the amount of Aβ 2-40 was decreased and the amount of Aβ 1-40 was conversely increased compared to controls. In cell free-conditioned media incubated over 48 h at 37°C, no decrease of the secreted Aβ 1-40 was observed, when CA-074 was added **(C)**. Treatment with the H^+^-ATPase inhibitor Bafilomycin A1 (Baf-A1) decreased the amount of Aβ 1-40 **(A)** and increased Aβ 2-40 **(B)** compared to controls. Interaction plots with CA-074 Me and Baf-A1 as independent variables suggested, that the increase of dependent variable Aβ 1-40 after CA-074 Me treatment was dependent on the level of Baf-A1 **(D)**, whereas no interaction was found for the dependent variable Aβ 2-40 **(E)**. Stains with Lysotracker **(F)** showed an increased particle size in H4 cells **(G)** and an increased particle mean fluorescence intensity (MFI) in human astrocytes **(H)** after the treatment with CA-074 Me compared to vehicle treated cells (con.). This effect was also observed in H4 CTSB –/– cells, which indicated a CatB-independent effect of CA-074 Me on the morphology of lysosomes. Statistics: **(A)** *n* = 5, ratio paired *T*-Test DMSO|CA-074 *t*_(4)_ = 3.395, *p* < 0.05, ratio paired *T*-Test DMSOICA-074Me *t*_(4)_ = 10.59, *p* < 0.001, **(B)** *n* = 4, ratio paired *T*-Test DMSO|CA-074 *t*_(3)_ = 8.544, *p* < 0.01, ratio paired *T*-Test DMSO|CA-074Me *t*_(4)_ = 6.868 *p* < 0.01, **(C)** *n* = 4, ratio paired *T*-Test *t*_(3)_ = 1.056, *p* > 0.05 **(D)** *n* = 3, Two-way ANOVA column *F* Baf-A1 (1, 8) = 68.54, *p* < 0.0001, row *F* CA-074 Me (1, 8) = 24.45, *p* < 0.0001, *F* interaction (1, 8) = 2.687, *p* < 0.05, **(E)** Two-way ANOVA F Baf-A1 (1, 8) = 89.70, *p* < 0.0001, *F* CA-074 Me (1, 8) = 7.734, *p* < 0.01, *F* interaction (1, 8) = 0.04241, *p* > 0.05, **(G)** Two-way ANOVA particle size *F* con. |CA-074 Me (1, 84) = 32.85, *p* < 0.0001, *F* CTSB +/−|CTSB –/– (1, 84) = 0.7853, *p* > 0.05, *F* interaction (1, 84) = 0.02116, *p* > 0.05, Two-way ANOVA particle MFI *F* con.|CA-074 Me (1, 80) = 5.279, *p* < 0.05, FCTSB +/−|CTSB –/– (1, 80) = 0.3039, *p* > 0.05, *F* interaction (1, 80) = 0.1415, *p* > 0.05, **(H)** unpaired *T*-Test particle size, *t*_(16)_ = 0.05915, *p* > 0.05, unpaired *T-*Test particle MFI, *t*_(15)_ = 2.351, *p* < 0.05. Tukey's *post-hoc* test was performed for multiple comparisons. Selected comparisons are indicated as follows ns *p* > 0.05, *p < 0.05; **p < 0.01; ***p < 0.001, and ****p < 0.0001). Each experiment was performed at least three times.

As the different effects of CatB inhibition on the Aβ profile of primary astrocytes or H4 cells might be due to different localizations of active CatB, H4 APP 751 cells and their supernatants alone were treated with the cell-impermeant CatB-inhibitor CA-074. In contrast to treatment with the cell-permeant Cat- inhibitor CA-074 Me, the treatment of H4 APP 751 cells with CA-074 over 48 h resulted not in an increase, but in a small decrease in secreted Aβ 1-40 to 82.2 ± 6.0% of control (Figure 3A; *T*-test *t*_(4)_ = 2.980, *p* < 0.05). The amount of Aβ 2-40 was decreased by CA-074 to a similar extent as CA-074 Me (Figure 3B). Incubation of cell-free, conditioned medium of H4 APP 751 cells with CA-074 over 48 h at 37°C did not change the amount of Aβ 1-x (Figure 3C). The CatB activity in cell culture supernatants varied substantially according to a CatB activity assay (data not shown). This indicated that the increase of total Aβ in H4 APP 751 cell culture supernatant was mediated by the inhibition of an intracellular enzyme, whilst the decrease of Aβ 2-40 after CA-074 Me and CA-074 treatment was probably mediated by the inhibition of a plasma membrane-associated enzyme. This is in accordance with our previous observation that Aβ peptide variants that were co-migrating with synthetic Aβ 1-40 in 2D SDS Urea-PAGE were detectable in lysates of cultured astrocytes, whereas Aβ peptide variants co-migrating with synthetic Aβ 2-40 could be detected in cell culture supernatants but not in cell lysates (Oberstein et al., 2015). Next, we investigated the effect of the vacuolar H^+^-ATPase inhibitor Bafilomycin A1 (Baf-A1), as Baf-A1 is known to inhibit the acidification of intracellular organelles such as lysosomes. Baf-A1 treatment resulted in a significant reduction of Aβ 1-x compared to controls in conditioned media of H4 APP 751 cells (Figures 3A,D), whereas the amount of Aβ 2-40 was increased (Figures 3D,E). Two-way ANOVA with Baf-A1 and CA-074 Me treatment as independent variables and Aβ 1-40 as dependent variable showed a significant interaction between the drug treatments (Figure 3D). The slopes of the corresponding interaction plot suggested that Baf-A1 treatment may reduce the degradation of Aβ 1-40 via CatB, as the slope steepness of the CA-074Me group was higher than the control group. Two-way ANOVA with Aβ 2-40 as the dependent variable showed no interaction between the Ca-074 Me and the Baf-A1 group (Figure 3E).

### Treatment With CA-074 Me Changed the Morphology of Lysosomes in Human Astrocytes and H4 Cells

As not only the involved enzymes but also the different cell compartment seemed to be crucial for the generation of the different Aβ variants, the effect of CA-074 Me on the morphology of lysosomes was studied (Figure 3F). Staining with LysoTracker® indicated an increased mean fluorescence intensity (MFI) of the detected particles in CA-074 Me treated human astrocytes (Figure 3H) and H4 CTSB –/+ cells compared to controls after 24 h (Figure 3G). In H4 cells, an increase in particle size was also detected after CA-074 Me treatment (Figure 3G). However, the increase of the particle MFI and size after CA-074 Me treatment was also detected in H4 CTSB –/– cells, and no differences in the MFI and particle volume were observed between CA-074 Me treated H4 CTSB +/− and H4 CTSB –/– cells (Figure 3H), which indicates that this effect was independent of the inhibition of CatB.

## Discussion

**Figure 4:**
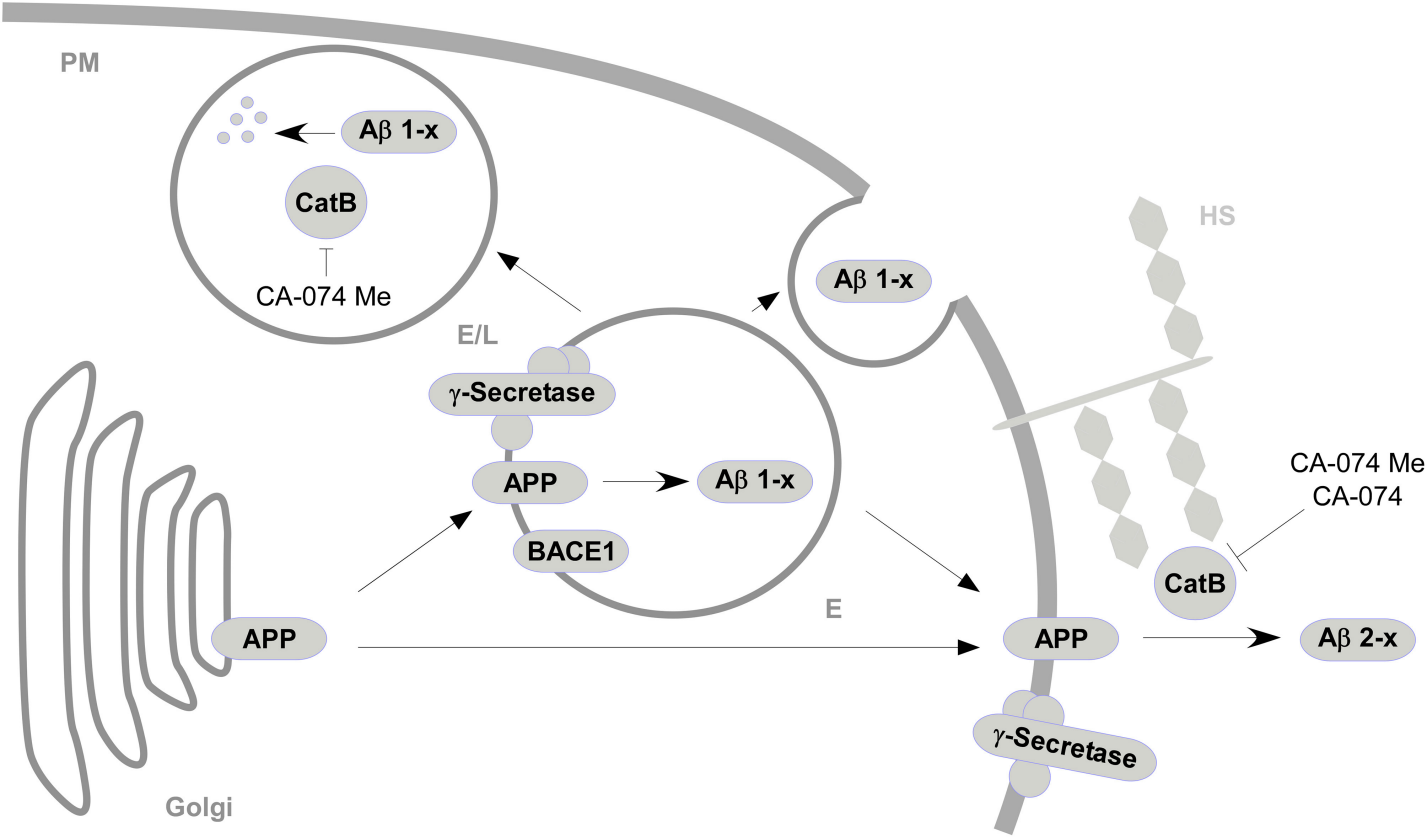
A simplified model for the sites of interaction between CatB, APP, and Aβ and the subsequent generation or degradation of Aβ based on the effects of CatB inhibition (|) with cell-permeable CA-074 Me and cell-impermeable CA-074. APP from the golgi apparatus is transported to acidified vesicles (E) or to the plasma membrane (PM) via vesicular transport (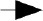). At the acificed vesicles, APP is processed (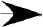) by BACE1 and γ-secretase to Aβ 1-x, which are secreted to the extracellular space or degraded in endosomes/lysosomes (E/L) by CatB. APP at the plasma membrane is processed to Aβ 2-x mediated by CatB presumably associated to heparan sulfate (HS) (Almeida et al., 2001).

The present study shows that the treatment of different astroglial cell cultures with the CatB-inhibitor CA-074 Me resulted in varying effects on the abundance of Aβ in supernatants depending on whether primary cells or a cell line was studied. Treatment with cell-impermeant CatB inhibitor CA-074 and the vacuolar H^+^-ATPase inhibitor Baf-A1 further indicated that the capacity of degrading Aβ 1-x and the generation of Aβ 2-x by CatB might be dependent on the different cellular localizations of active CatB (Figure 4).

The amount of secreted Aβ 2-40, but not that of Aβ 1-40 in supernatants of cultured primary chicken or human astrocytes was reduced by treatment with CatB inhibitors. Thus, our data indicate that CatB is probably involved in the production of the N-terminally truncated Aβ 2-x. However, small amounts of these N-terminally modified Aβ peptide variants were detected, even after CatB inhibition with CA-074 Me. The observed decrease in Aβ 2-40 after CatB inhibition in cultured primary astrocytes is in accordance with a previous report, showing that purified CatB can cleave peptide substrates flanking the β-secretase site within APP between Lys and Met and between Asp and Ala (Schechter and Ziv, 2011). Bien et al. (2012) and we have previously reported that the production of Aβ 2-x was independent of BACE1 (Bien et al., 2012; Oberstein et al., 2015). Butler et al. (2011) reported that the activity of CatB was not influenced by commonly used BACE1 inhibitors. However, CatB does not seem to be the only endopeptidase, which is capable of cleaving APP N-terminal to Ala2 of the Aβ-Sequence. Previous reports state that Aβ 2-x can also be produced by the endoproteolytic activity of meprin β (Bien et al., 2012; Schonherr et al., 2016). Furthermore, Aminopeptidases, such as Aminopeptidase A, may be involved in the N-terminal truncation of Aβ following the primary cleavage of APP by BACE1 (Saido, 1998; Sevalle et al., 2009). Additionally, it has been reported that CatB cleaves APP-derived substrates at Asp and isoAsp as assessed by cell-free enzymatic assays (Bohme et al., 2008). However, in our cell culture model, the inhibition of CatB by CA-074 Me resulted in a slight, non-significant decrease of Aβ 1-40 in primary chick or human astrocytes. Given the differences in the sequence of APP between chickens and humans, these might alter the specificity and selectivity of CatB and other proteases to perform cleavage of APP at the beta-site. In our study, however, Aβ 1-37 was the only detected Aβ peptide variant that was changed in a statistically significantly different manner by CA-074Me-treatment in human astrocytes compared to chicken astrocytes. Since there was no difference in the levels of Aβ 1-40 and Aβ 1-42, we speculate that the difference observed for Aβ 1-37 was not due to cleavage of the beta-site of APP by CatB. Collectively, our data suggest that CatB is involved in the production of N-terminally truncated Aβ 2-x in primary astrocytes but not in the generation of Aβ 1-x. In H4 APP751 cells, the N-terminally truncated Aβ 2-40 was also reduced after inhibition with CA-074Me or with CA-074 as well as after the deletion of CTSB via CRISPR/Cas9 technology.

In contrast to the observations from primary cell culture experiments, CA-074 Me treatment and deletion of CTSB via CRISPR/Cas9 technology in H4 APP751 cells led to a significant increase of Aβ 1-x. Thus, it appears that CatB takes part in the degradation of Aβ 1-x in H4 cells overexpressing APP. In line with that, Mueller-Steiner et al. (2006) reported that CatB reduced Aβ levels in hippocampal CA1 pyramidal neurons of hAPP mice. In our study, the vacuolar H^+^-ATPase inhibitor Baf-A1 reduced the amounts of Aβ 1-40, Aβ 1-42, and Aβ 1-38 in conditioned media, and simultaneous treatment with CA-074 Me suggested that the CA-074 Me-mediated increase of Aβ 1-40 was dependent on acidified compartments. This is in accordance with the reported dipeptidyl carboxypeptidase activity of CatB, which has its optimum at pH 5.0, i.e., the pH of late endosomes and lysosomes (Mach et al., 1994; Almeida et al., 2001; Mueller-Steiner et al., 2006; Butler et al., 2011; Wang et al., 2012). As we did not observe any increase of Aβ 1-x after treatment with the cell-impermeant inhibitor CA-074 in supernatants of H4 cells, we concluded that Aβ 1-x was not degraded extracellularly by CatB. It appears that CA-074 did not enter lysosomes via extensive pinocytosis/fluid phase endocytosis. This is in line with the report of Bogyo et al. (2000) who stated that the derivatives of CA-074, CA-074b and MB-074, were not capable of entering the lysosome and inhibiting the target. In summary, the findings suggest that lysosomal CatB might be involved in Aβ 1-x degradation. In our cell culture model, all detected Aβ 1-x variants, including Aβ 1-42 and Aβ 1-38, were similarly increased after inhibition of CatB or deletion of CTSB. In cell-free assays, the carboxypeptidase activity of CatB did not produce C-terminally truncated Aβ peptide variants from Aβ 1-42 that were shorter than 38 or 37 amino acids (Mackay et al., 1997; Mueller-Steiner et al., 2006). Correspondingly, Butler et al. (2011) and Hwang et al. (2019) reported that the pharmacological modulation of lysosomes and thereby increased levels and activity of CatB resulted in a decrease of Aβ 42 and an increase of Aβ 38 in APPswe/PSEN1dE9 mice. We can only speculate, why we did not observe a shift toward Aβ 1-42 after CatB inhibition with CA-074 Me or deletion of CTSB in H4 APP751 cells in our study. One possible explanation might be that in our model other proteases rapidly degrade these CatB-produced, C-truncated Aβ fragments further into smaller fragments. A variety of proteases have been implicated in the degradation of Aβ, like neprilysin, plasmin, and insulin degrading enzyme (Howell et al., 1995; Qiu et al., 1998; Van Nostrand and Porter, 1999). Recently, Kidana et al. (2018) reported that kallikrein-related peptidase 7, a serine protease, contributes to the degradation of Aβ in astrocytes. Additionally, the overexpression of APP751 might also lead to an aberrant trafficking and/or C-Terminal truncation. The work of Butler et al. (2011) and Hwang et al. (2019) suffers from a similar dilemma, as they use transgenic animal models: The use of the so-called Swedish mutation of APP and the PSEN1dE9 mutation in both of the animal models should result in a pattern of secreted Aβ with abnormal high percentages of Aβ 1-42, because the Swedish mutations increases the affinity of APP for BACE1 cleavage and the PSEN1dE9 mutations reduces the inherent carboxypeptidase activity of the γ-secretase. Conversely, the lower amount of APP and the unrestricted carboxypeptidase activity of the γ-secretase could be the reason why lysosomal CatB was apparently not involved in the degradation of Aβ in primary chicken and human astrocytes. However, the increased amount of Aβ 1-40 in supernatants of CA-074 Me-treated, untransfected H4 cells indicates that the overexpression of APP is not sufficient to explain the differences between H4 cells and primary astrocytes.

In contrast to Aβ 1-x, the decrease of Aβ 2-x in conditioned media after treatment of cultured cells with either CA-074Me or CA-074 and its increase upon Baf-A1 treatment suggests that Aβ 2-x is preferentially generated outside lysosomal compartments, i.e., extracellularly or near the plasma membrane in non-acidic cellular compartments. It has been reported before that CatB is frequently redistributed to the plasma membrane (Frosch et al., 1999; Cavallo-Medved and Sloane, 2003). CatB must then be stabilized by heparin sulfate at the cell surface, as otherwise it loses it proteolytic activity (Taralp et al., 1995; Almeida et al., 2001). Congruently, CatB activity in cell free medium was mostly below the LLOD and subject to great variances in the CatB activity assay. An abberant extracellular distribution of CatB has been described for AD near senile plaques (Cataldo et al., 1990). It has recently been hypothesized that leakage of lysosomal CatB into the cytosol contributes to neurodegeneration and behavioral deficits in AD and traumatic brain injury (Hook et al., 2020). The presumed generation of Aβ 2-x by non-lysosomal CatB in astrocytes in this study supports the hypothesis of deleterious effects of non-lysosomal CatB, as Aβ 2-40 is potentially associated with CAA in AD (Gkanatsiou et al., 2019).

Our study indicates that the decrease of Aβ 2-40 after CA-074 Me treatment might be mediated by both CatB-dependent and CatB-independent mechanisms: CA-074 has been reported to inhibit CatB more selectively than CA-074 Me (Bogyo et al., 2000; Montaser et al., 2002). This is in favor of a CatB-dependent decrease of Aβ 2-40 after inhibitor treatment, as CA-074 treatment lowered the amount of Aβ 2-40 to the same extent as CA-074 Me in our study. Additionally, Aβ 2-40 in conditioned media of H4 APP 751 CTSB –/– cells was moderately decreased in comparison to H4 APP 751 cells. However, CA-074 Me had an additional effect and further decreased the amount of Aβ 2-40 in conditioned medium of H4 APP 751 CTSB –/– cells. This additional reduction in Aβ 2-40, which was apparently not directly related to CatB inhibition, might be the consequence of altered lysosomal function and trafficking. In H4 cells LysoTracker organelle dyes revealed enlarged vesicles after the CA-074 Me treatment. This effect was observed even after the deletion of CTSB in H4 cells. CA-074 Me is considered to be a specific CatB inhibitor (Murata et al., 1991; Buttle et al., 1992). Nevertheless, there have been previous reports of CA-074 Me effects that were independent of CatB inhibition. These might possibly be due to the methylation of the proline carboxyl group facilitating binding to cathepsins other than CatB, like cathepsin L, or increasing the lysosomal membrane integrity (Bogyo et al., 2000; Montaser et al., 2002; Mihalik et al., 2004; Xu et al., 2016). In accordance with previous studies from Hook et al. (2010) and Mueller-Steiner et al. (2006), the treatment with CA-074 Me and E64d, did not alter the cellular levels of the different APP isoforms and BACE1 in our study.

This study indicates that the observed adverse and positive effects of CatB and its inhibitors may depend on the sites of interaction with APP and its metabolites. The use of techniques like fluorescence (life cell) imaging, activity-based probes and small molecule inhibitors with different effects on the endo- and exopeptidase activity of CatB will be helpful in further elucidating the sites of interaction between CatB and APP both *in vitro* and ultimately *in vivo* and possibly contribute to the development of suitable drugs.

## Conclusion

Lysosomal CatB seems to be involved the degradation of Aβ 1-x in neuroglioma cell culture but not in primary astrocytes. The generation of N-terminally truncated Aβ 2-x in astrocytes, however seemed to be mediated by plasma-membrane associated CatB.

## Data Availability Statement

The raw data supporting the conclusions of this article will be made available by the authors, without undue reservation.

## Author Contributions

TO designed the study, performed experiments, analyzed the data, and drafted the manuscript. JU performed experiments and contributed to revision of the manuscript. JK and JM provided reagents and contributed to the interpretation of findings and revision of the manuscript. PS, PL, JW, and HK contributed to the interpretation of findings and revision of the manuscript. All the authors read and approved the final manuscript.

## Conflict of Interest

The authors declare that the research was conducted in the absence of any commercial or financial relationships that could be construed as a potential conflict of interest.

## Abbreviations

AD: Alzheimer's disease
Aβ: Amyloid beta
ADAM: A Disintegrin and Metalloproteinase
APP: Amyloid Precursor Protein
BACE1: beta-site APP cleaving enzyme 1
Baf-A1: Bafilomycin A1
CatB: cathepsin B
CRISPR: Clustered Regularly Interspaced Short Palindromic Repeats
CTSB: gene enconding CatB
con.: vehicle treated controls
DMEM: Dulbecco's Modified Eagle's Medium
DMSO: Dimethylsulfoxid
d.f.: degrees of freedom, HEPES, 4-(2-hydroxyethyl)-1-piperazineethanesulfonic acid
LLOD: lower limit of detection
n: sample size
mAb 82E1: monoclonal antibody, which specifically recognizes Aβ 1-x
pAb p77: polyclonal antibody, which specifically recognizes Aβ 2-x
PAGE: Polyacrylamide gel electrophoresis
SDS: sodium dodecyl sulfate.
