# Supplementary figures and images for: The Role of Cathepsin B in the Degradation of Aβ and in the Production of Aβ Peptides Starting With Ala2 in Cultured Astrocytes
Source: Front Mol Neurosci. 2021 Jan 12;13:615740. doi: 10.3389/fnmol.2020.615740 (PMC7836726; doi:10.3389/fnmol.2020.615740)

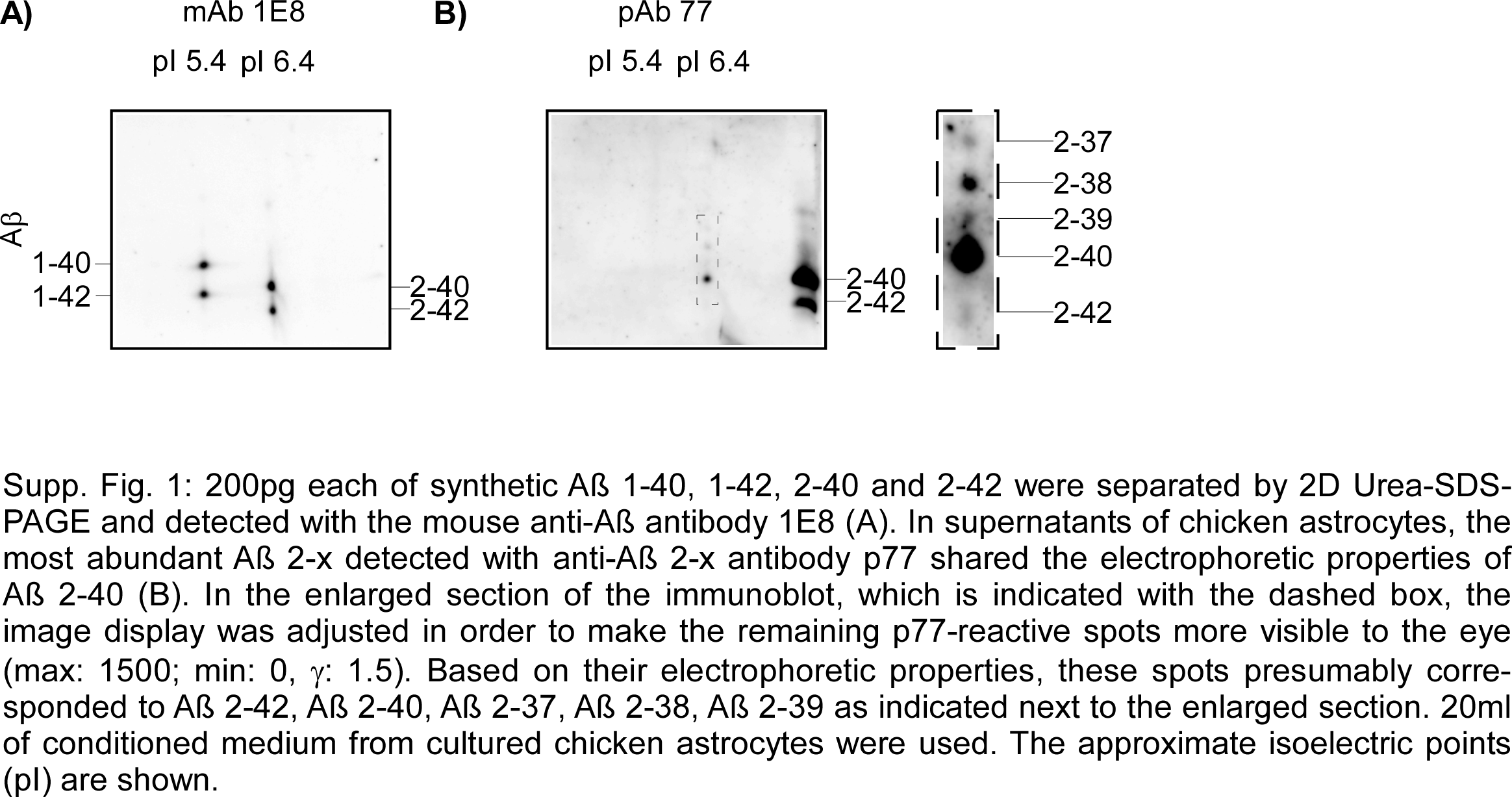

Supplement: Supplementary file 1 [file Image_1.TIF]

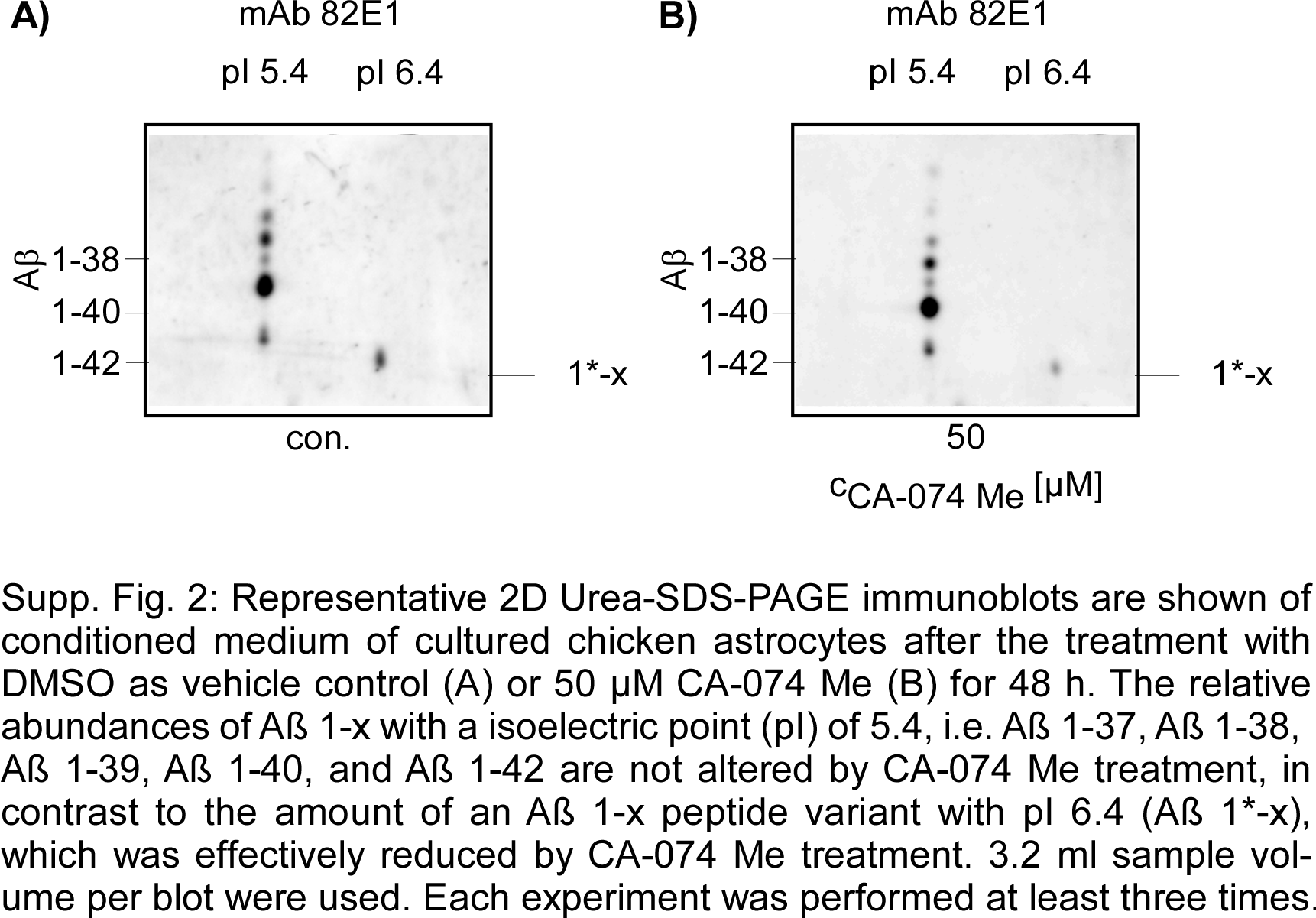

Supplement: Supplementary file 2 [file Image_2.TIF]

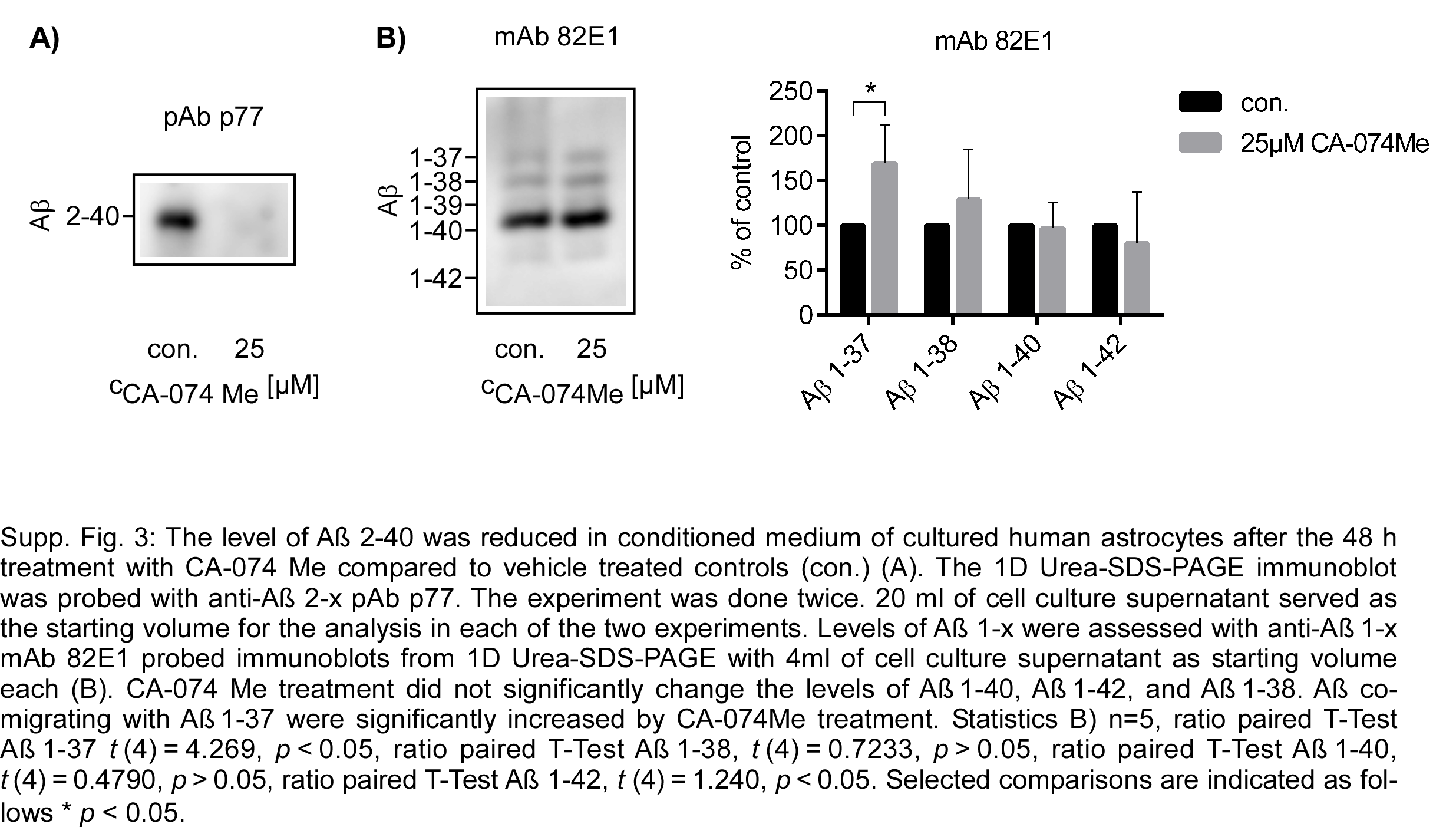

Supplement: Supplementary file 3 [file Image_3.TIF]

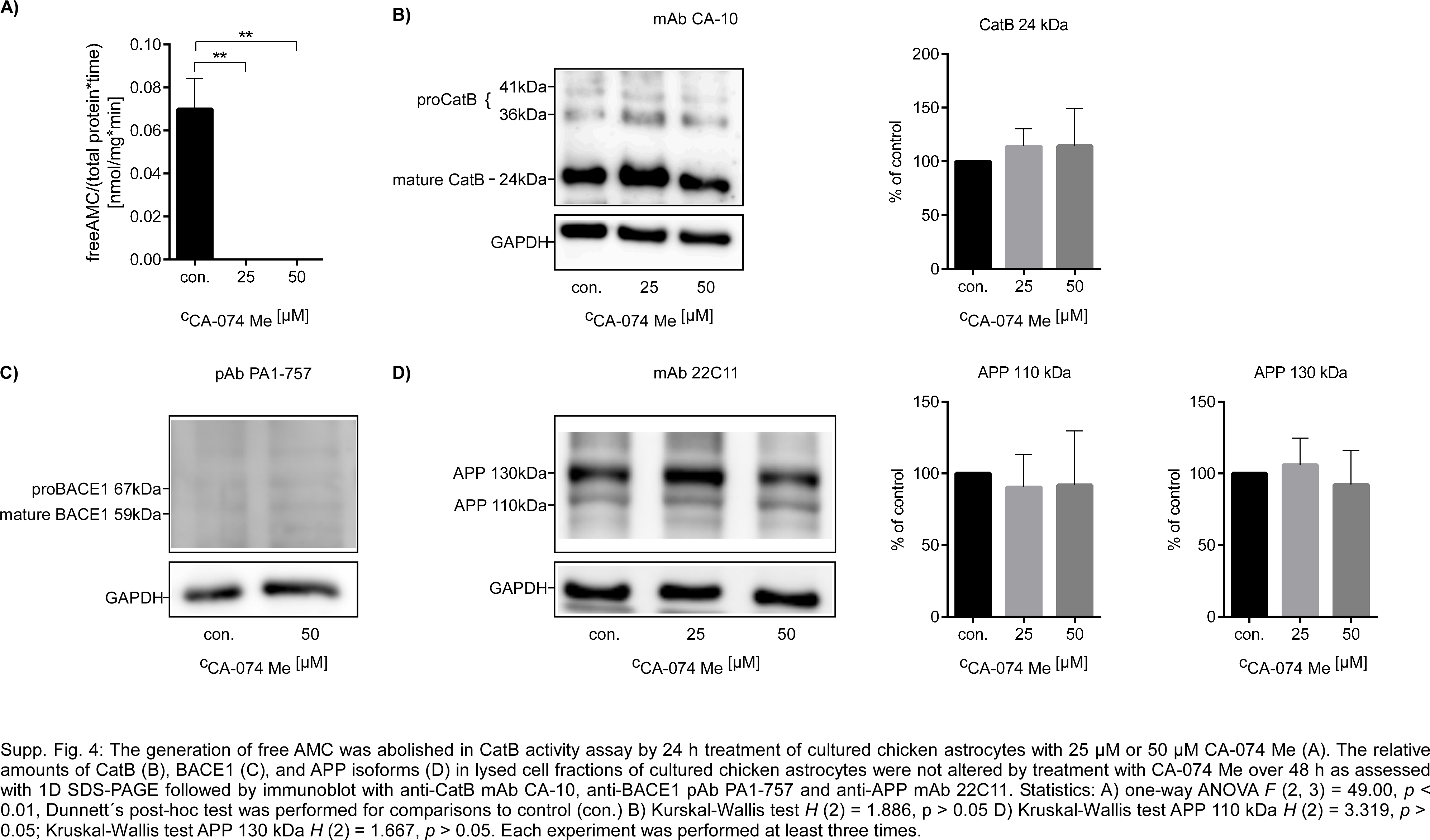

Supplement: Supplementary file 4 [file Image_4.TIF]

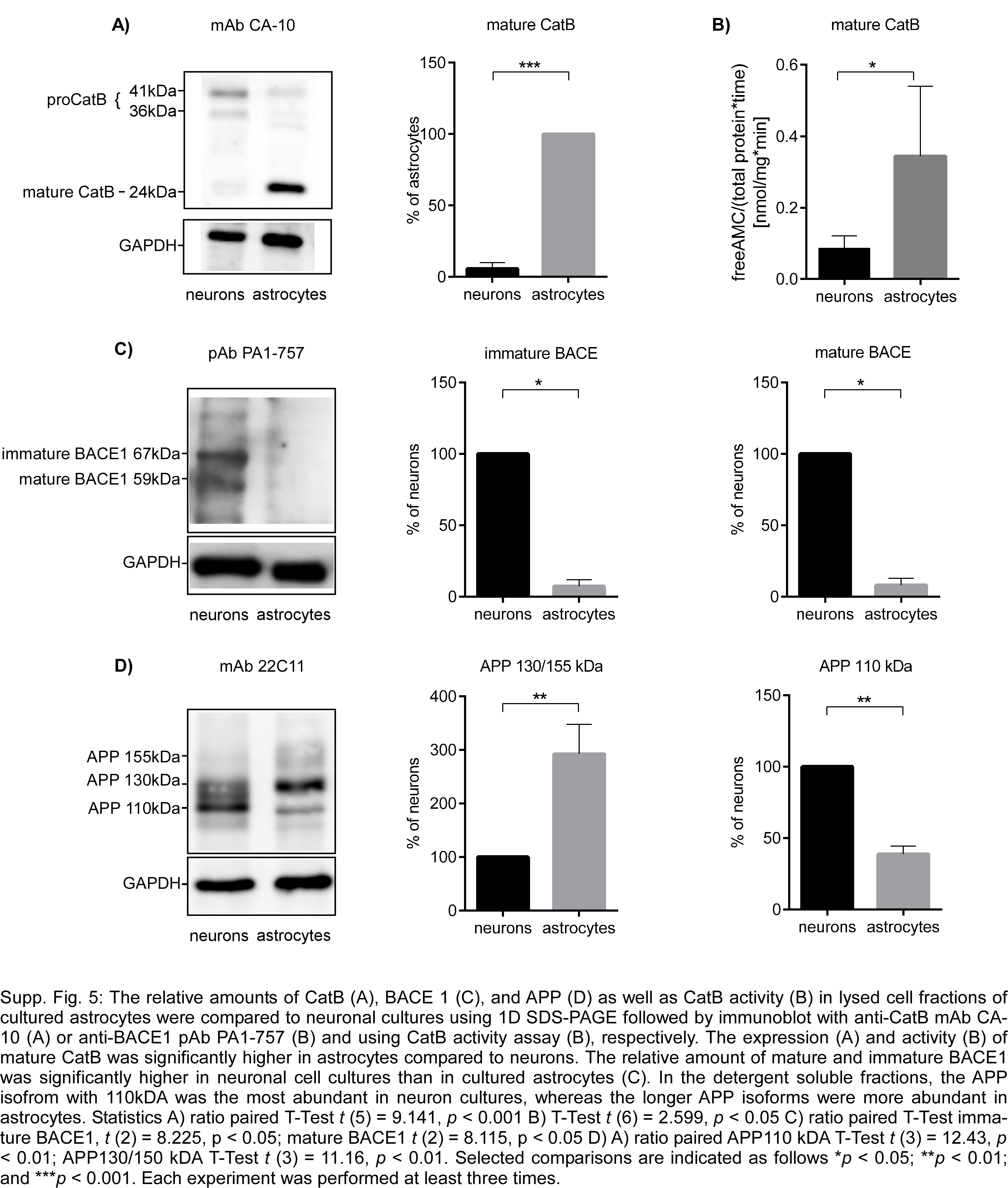

Supplement: Supplementary file 5 [file Image_5.TIF]

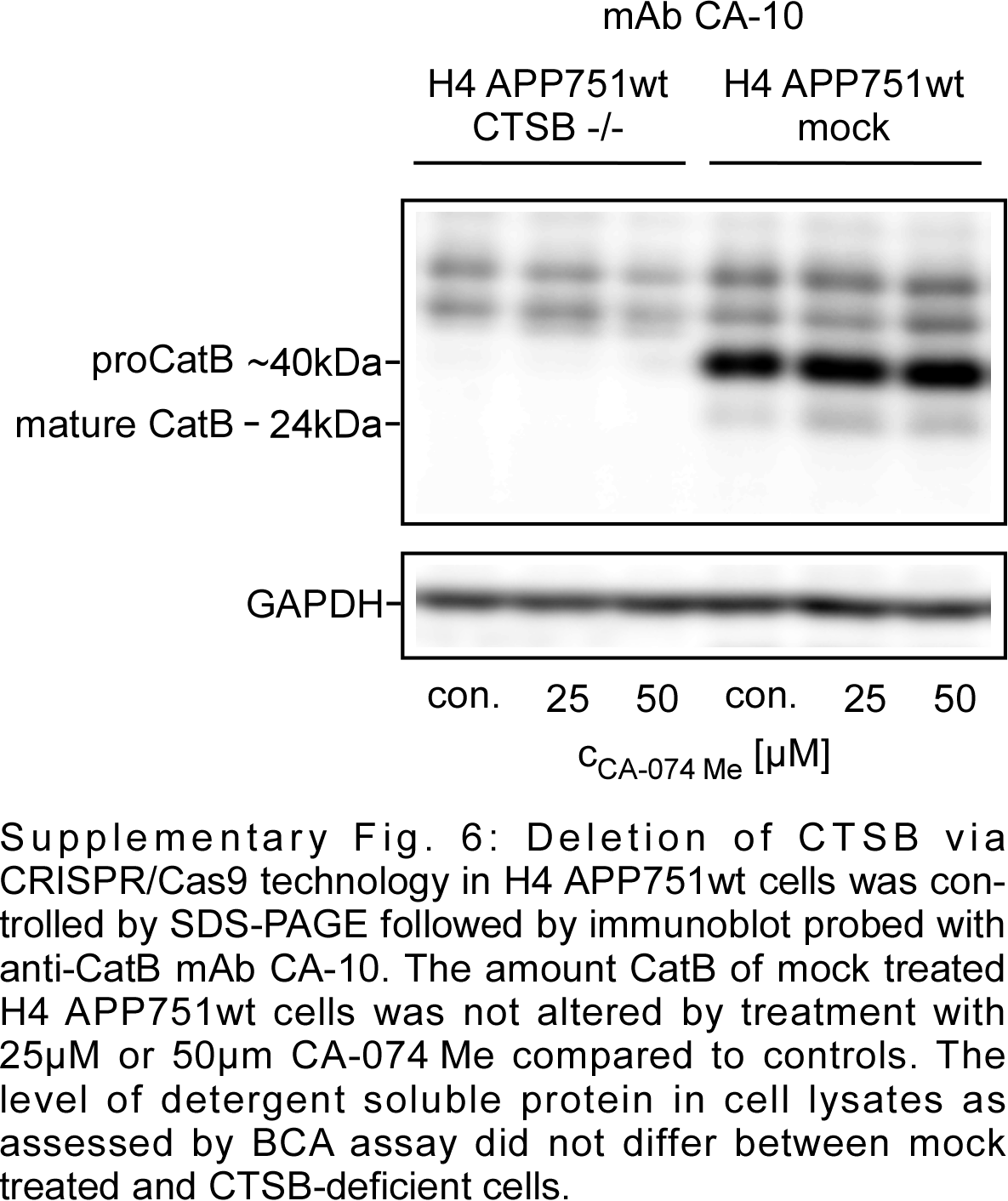

Supplement: Supplementary file 6 [file Image_6.TIF]

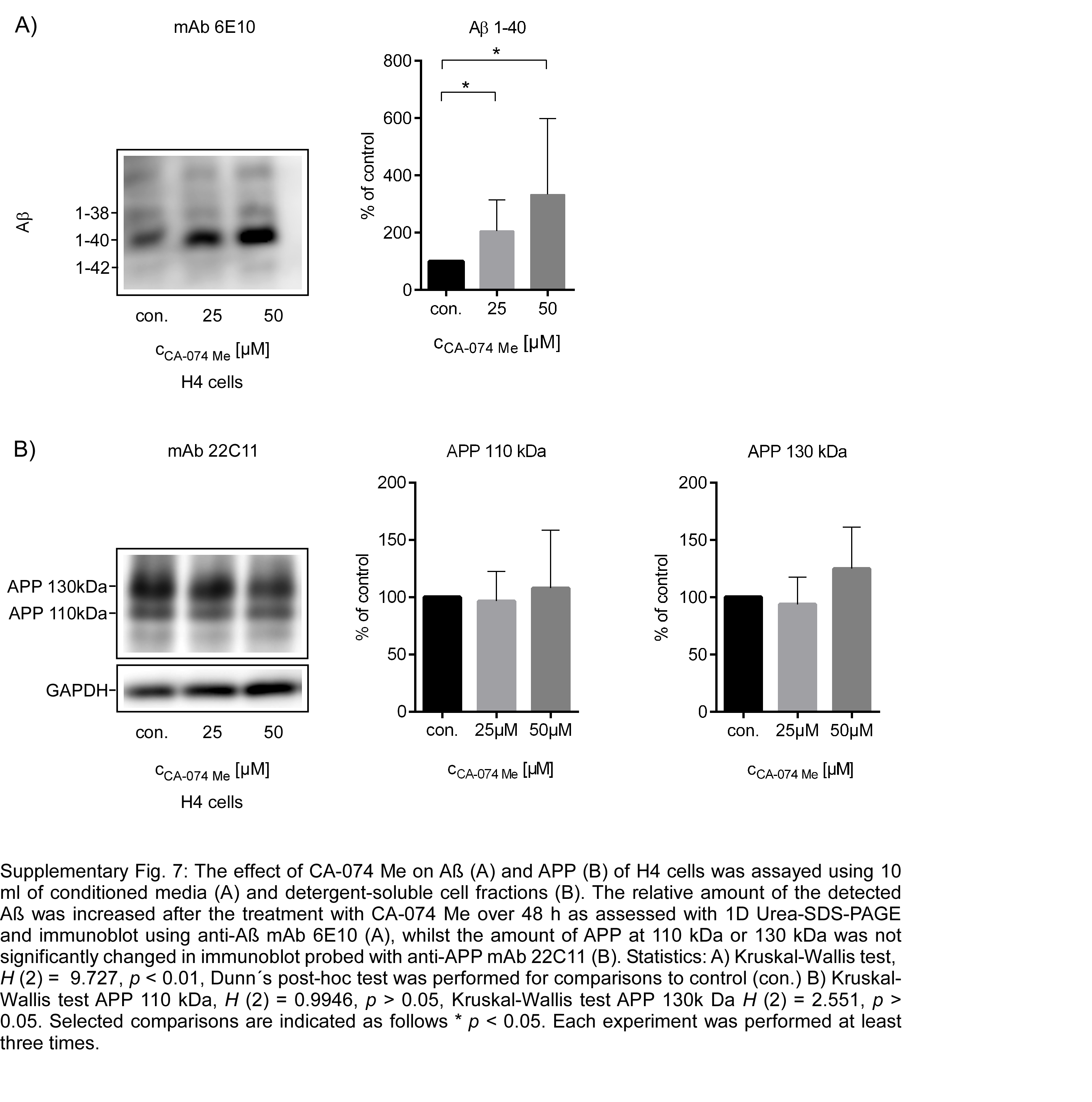

Supplement: Supplementary file 7 [file Image_7.TIF]
